# MCM7 promotes cancer progression through cyclin D1-dependent signaling and serves as a prognostic marker for patients with hepatocellular carcinoma

**DOI:** 10.1038/cddis.2016.352

**Published:** 2017-02-09

**Authors:** Kai Qu, Zhixin Wang, Haining Fan, Juan Li, Jie Liu, Pingping Li, Zheyong Liang, Hongli An, Yina Jiang, Qiushi Lin, Xiaoqun Dong, Peijun Liu, Chang Liu

**Affiliations:** 1 Department of Hepatobiliary Surgery, The First Affiliated Hospital of Xi'an Jiaotong University, Xi'an 710061, Shaanxi Province, China; 2 Department of Hepatopancreatobiliary Surgery, Affiliated Hospital of Qinghai University, Xining 810001, Qinghai Province, China; 3 Center for Translation Medicine, The First Affiliated Hospital of Xi'an Jiaotong University, Xi'an 710061, Shaanxi Province, China; 4 Department of Pathology, The First Affiliated Hospital of Xi'an Jiaotong University, Xi'an 710061, Shaanxi Province, China; 5 Department of Internal Medicine, College of Medicine, The University of Oklahoma Health Sciences Center, Oklahoma City, OK 73104, USA

## Abstract

DNA replication is a central procedure of cell proliferation, whereas aberrant DNA replication is indicated to be a driving force of oncogenesis. Minichromosome maintenance complex component 7 (MCM7) plays an essential role in initiating DNA replication. To investigate the potential oncogenic properties and prognostic value of MCM7 in hepatocellular carcinoma (HCC), we conducted immunohistochemistry staining of MCM7 in 153 HCC samples and found that MCM7 high expression level was associated with worse overall survival (OS) of HCC patients. Mechanistically, knockdown of MCM7 significantly inhibited cellular proliferation *in vitro* and HCC tumorigenicity *in vivo*. Cyclin D1 was proved to be regulated by MCM7–MAPK signaling pathway. Clinically, high expression of both MCM7 and cyclin D1 exhibited a relatively high sensitivity and specificity to predict worse outcome of HCC patients. Taken together, our results suggest that MCM7–cyclin D1 pathway may participate in cancer progression and serve as a biomarker for prognosis in HCC.

Hepatocellular carcinoma (HCC) is the fifth most common cancer worldwide, and patient population in China alone accounts for more than half of the total cases mainly because of hepatitis B virus (HBV) infection.^1, 2^ As one of the leading causes of mortality, HCC is usually diagnosed at an advanced stage and the patients would die of the disease within several months.^3^ Despite improvements in surgical techniques and standard-of-care treatments, the prognosis of HCC remains very poor.^4, 5^ Traditional clinicopathological features such as tumor stage, histological grade and serum *α*-fetoprotein (AFP) level seem to insufficiently predict clinical outcome in HCC patients.^6^ Therefore, a thorough understanding of the underlying mechanisms regarding tumor progression is critical for identifying novel discriminatory biomarkers and developing effective therapeutic targets for HCC patients.

Enabling replicative immortality, as an important hallmark of cancer cells,^7, 8^ requires unlimited potential in DNA replication. At the initiation stage of this process, the minichromosome maintenance (MCM) complex is considered as a critical first step of formation of pre-replication complex that melts origin DNA and unwinds replication forks.^9, 10^ In almost all eukaryotes, the MCM complex is composed of six highly conserved MCM proteins, namely MCM2–7 (minichromosome maintenance complex components 2–7).^11^ Several MCM proteins are recently found to be tightly associated with tumorigenesis.^12, 13, 14, 15, 16, 17, 18, 19, 20^ Among of them, MCM7, an important subunit of the presumed heteromeric MCM helicase, is reported to be involved in tumor formation and progression, and is considered as a potential biomarker in a variety of human malignancies.^17, 18, 20, 21^ In HCC, elevated expression of MCM7 was found in nearly half of tumor samples, and significantly correlated with intrahepatic metastasis and vascular invasion.^20^ In addition, MCM7 expression was also observed to be inhibited by sorafenib and other anti-HCC therapies.^20, 22, 23^ Although accumulating evidence has suggested a critical role of MCM7 in liver carcinogenesis, the molecular mechanisms underlying pro-HCC functions and prognostic implication of MCM7 remain to be elucidated.

The present study aims to demonstrate whether MCM7 plays a key role in promoting HCC progression and to explore underlying molecular mechanisms. Furthermore, we are also interested in evaluating the prognostic and predictive values of MCM7 along with its downstream signaling molecules in HCC patients.

## Results

### Overexpressed MCM7 is associated with more aggressive malignant characteristics of HCC tumors

**Figure 1:**
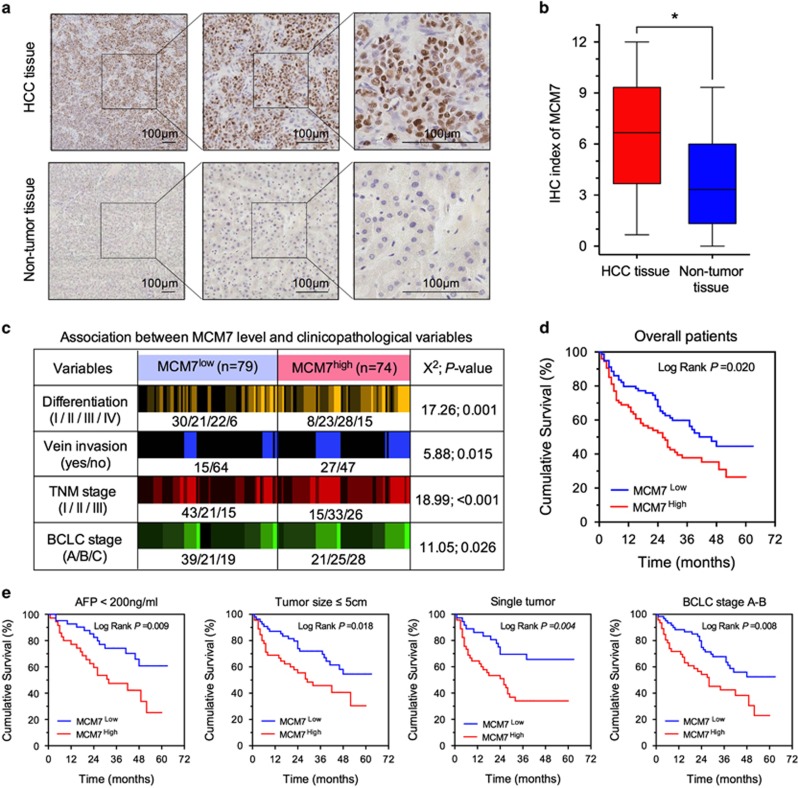
MCM7 was overexpressed in HCC and association with patients' survival. (**a**) Representative IHC staining images of MCM7 expression in human HCC and nontumor tissues, bar=100 *μ*m. (**b**) The IHC index of MCM7 in HCC and nontumor tissues. (**c**) Association between MCM7 expression and clinicopathological variables in HCC patients. (**d** and **e**) The cumulative overall survival differences between patients with high and low MCM7 expression in all patients (**d**) and different subgroups (**e**). *P<0.05

Exploration using publicly available data sets (http://kmplot.com/analysis/) suggested that higher MCM7 mRNA expression was significantly correlated with a worse overall survival (OS) time in breast, gastric and lung cancer patients (all *P*<0.01, Supplementary Figure S1). To gain insight into the clinical signature of MCM7 in HCC, we examined the expression levels of MCM7 in 153 paired HCC tumor tissues and adjacent normal liver samples by immunohistochemistry (IHC) staining. As shown in Figure 1a, positive staining of MCM7 was very frequently seen in the nuclei of the HCC cells. In contrast, very few positive cells were detected in their paired nontumor tissues. The IHC index of MCM7 in HCC tumor tissues is much higher than nontumor tissues (*P*<0.05, Figure 1b), suggesting that MCM7 is mainly overexpressed in HCC. To explore the association between MCM7 expression level and clinicopathological features of HCC, we dichotomized 153 HCC patients into two subgroups, namely MCM7^low^ and MCM7^high^. Subjects were considered as MCM7^low^ if their IHC index of MCM7 was within the range of 1 to 6, and MCM7^high^ if the IHC index was between 7 and 12. As shown in Figure 1c, higher MCM7 expression was found to be associated with more aggressive malignant characteristics, such as poorer tumor differentiation (*P*=0.001), higher frequency of vein invasion (*P*=0.015) and more advanced tumor stage (TNM staging: *P*<0.001 and BCLC staging: *P*=0.026).

### Higher MCM7 expression is associated with worse survival in HCC patients

Next, we performed univariate and multivariate Cox regression analyses to assess the individual and incremental prognostic values of both MCM7 and clinicopathological parameters in HCC patients (Supplementary Table S1). In addition to the classical clinical prognostic factors, MCM7 expression was an independent factor affecting patients' survival in HCC (HR (95% CI): 1.64 (1.07–2.51), *P*=0.023) (Supplementary Table S1). We further investigated the influence of MCM7 expression level on OS time using Kaplan–Meier method. We found that the HCC patients with higher MCM7 expression always had shorter median OS as compared with those with lower MCM7 expression (log rank *P*=0.020, Figure 1d).

Similarly, consistent association between MCM7 expression level and clinical outcome was observed in HCC patients who were stratified by demographic and clinicopathological characteristics. Interestingly, the death risk of low MCM7 expression on OS was found to be smaller in those HCC patients with less malignant characteristics, such as low AFP level (< 200 ng/ml), small tumor size, single tumor number and early BCLC stage (all *P*⩽0.036, Table 1). Kaplan–Meier analysis further confirmed a long median OS time in those patients with low MCM7 expression in the above-mentioned stratified subgroups (all *P*⩽0.018, Figure 1e).

### Downregulation of MCM7 suppresses HCC cell proliferation *in vitro*

**Figure 2:**
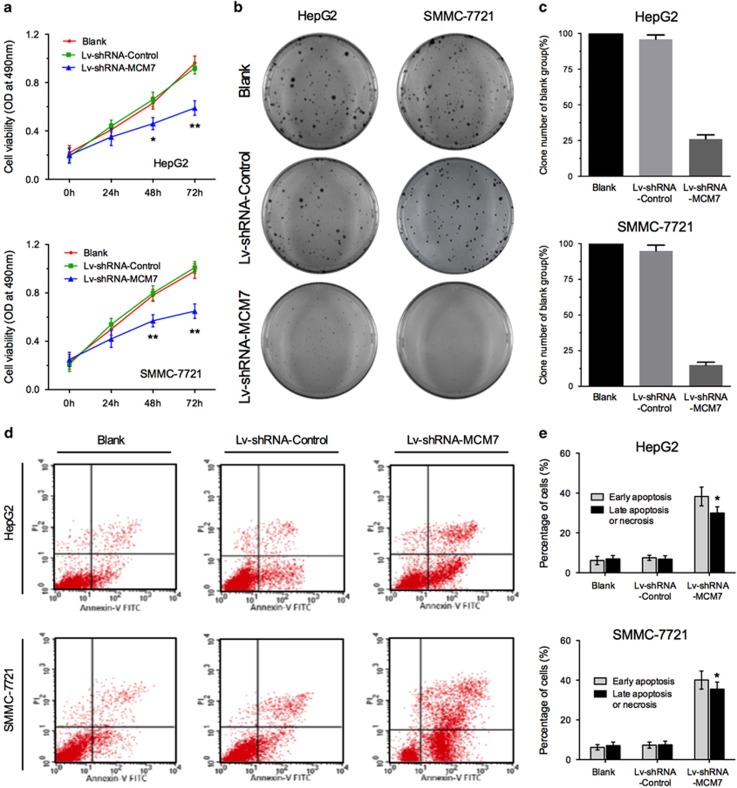
Knockdown of MCM7 inhibited proliferation of HCC cells. (**a**) MTT assay of HCC cells transfected by Lv-shRNA-MCM7 or Lv-shRNA-Control. (**b** and **c**) Colony-forming ability of HCC cells transfected by Lv-shRNA-MCM7 or Lv-shRNA-Control. (**d** and **e**) Flow cytometry analysis for apoptosis in HCC cells after 72 h of transduction. Data are represented as mean±S.E.M. from three independent experiments. *P<0.05; **P<0.01; ***P<0.001

To further explore the molecular mechanisms underlying tumor suppression induced by low MCM7 expression in HCC, MCM7 knockdown in two HCC cell lines, HepG2 and SMMC-7721, was conducted using lentiviral vector transduction system (Lv-shRNA-MCM7 and Lv-shRNA-Control). Our data showed that downregulation of MCM7 significantly suppressed cell viability in HepG2 and SMMC-7721 cells (Figure 2a). In addition, the observation from colony formation assay also confirmed the suppression of cell growth in HCC cells with MCM7 downregulation (Figures 2b and c). Furthermore, we performed Annexin V-FITC/PI flow cytometry to assess the effect of MCM7 downregulation on tumor cell apoptosis. The results revealed a significant induction of both early and late phases of apoptosis in HCC cells with MCM7 downregulation (Figures 2d and e).

### Downregulation of MCM7 restrains cell cycle progression mainly by suppressing cyclin D1 expression

**Figure 3:**
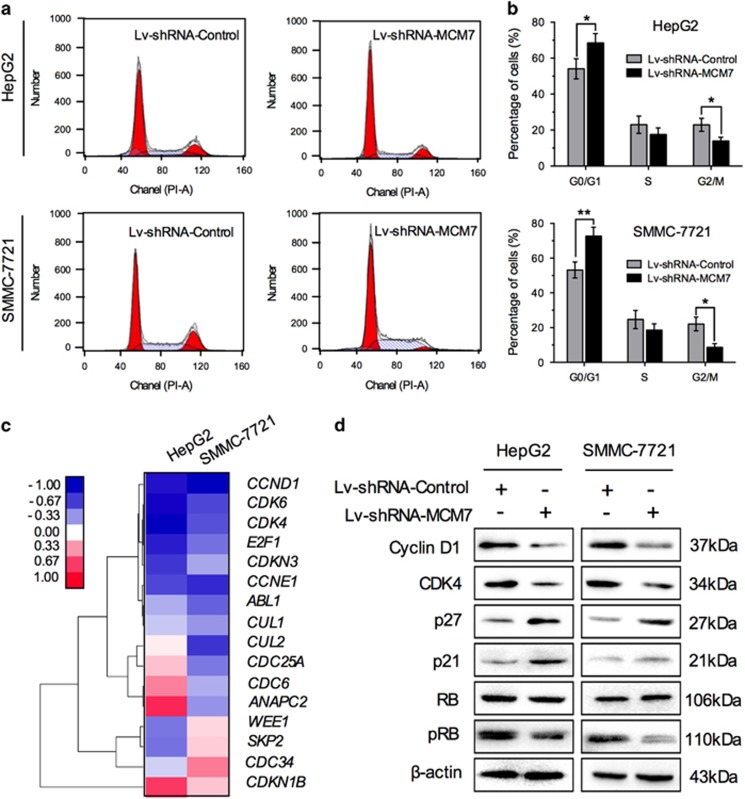
Downregulation of MCM7 restrains cell cycle progression by suppressing cyclin D1 expression. (**a** and **b**) Cell cycle analysis of HCC cells transfected by Lv-shRNA-MCM7 or Lv-shRNA-Control. (**c**) A total of 16 cell cycle-related genes were screened after lentiviral vector transduction. (**d**) Western blot analysis of cyclin D1, CDK4, p27, p21, total RB and p-RB in HCC cells after lentiviral vector transduction. Data are represented as mean±S.E.M. from three independent experiments. *P<0.05; **P<0.01

The essential role of MCM7 in DNA replication has been demonstrated by previous studies.^11^ However, the effect of MCM7 modulation on cell cycle in HCC cells has rarely been addressed. In the present study, we detected cell cycle status in HCC cells with MCM7 downregulation by flow cytometric assay. As shown in Figures 3a and b, the percentage of G0/G1 phase was significantly increased in Lv-shRNA-MCM7-transfected cells as compared with empty vector (Lv-shRNA-Control)-transfected cells. These results suggested that inhibition of MCM7 restrained cell cycle progression and mainly induced G1 arrest in HCC cells.

It is well known that numerous cell cycle genes are involved in regulation of G1/S transition.^24^ To find out the regulatory pathway that is affected by MCM7 and consequently causes G1 arrest, we screened gene expression profile including 16 G1/S-regulatory genes using the real-time reverse transcription PCR (qRT-PCR) measurement. The expression of *cyclin D1* (*CCND1*) was shown to decrease at approximately twofold in HCC cells with MCM7 downregulation (Figure 3c). In addition, several downstream molecules of cyclin D1, such as cyclin D-dependent kinase CDK6 and CDK4, as well as transcription factor E2F1,^25^ were also affected by MCM7 downregulation (Figure 3c). Western blot assay further confirmed the decreased levels of cyclin D1 and its downstream molecules CDK4 and phosphorylated retinoblastoma protein RB (pRB) in Lv-shRNA-MCM7-transfected cells (Figure 3d). Meanwhile, the cyclin D1 inhibitors, p21 and p27, were shown to be upregulated by MCM7 downregulation (Figure 3d), suggesting a critical role of cyclin D1 in MCM7-mediated cell cycle progression.

### Knockdown of MCM7 downregulates cyclin D1 expression via suppressing MAPK signaling activity

**Figure 4:**
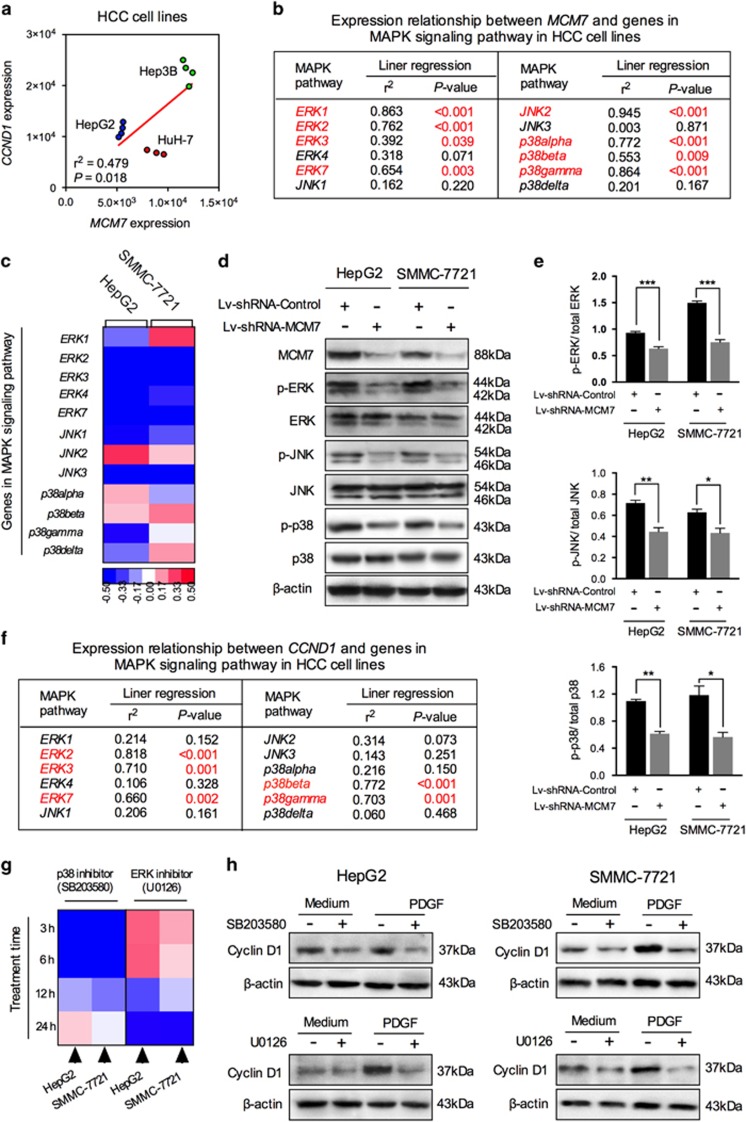
MAPK signaling participated in regulating the MCM7–cyclin D1 signaling in HCC cells. (**a**) Correlation of *MCM7* with *CCND1* gene expression in three HCC cell lines. (**b**) Correlation of *MCM7* with 12 MAPK pathway member gene expression. (**c**) Heat map of changes in the expression of 12 MAPK pathway member genes after lentiviral vector transduction. Western blot analysis (**d**) and corresponding bar graphs (**e**) validated the alteration in the activity of three major MAPK members (ERK, JNK and p38) in HCC cells. (**f**) Correlation of *CCND1* with 12 MAPK pathway member gene expression. (**g**) Heat map of changes in the expression of *CCND1* after pretreatment with SB203580 (p38 inhibitor) or U0126 (ERK inhibitor) at different time points (3, 6, 12 and 24 h).; (**h**) Western blot analysis for cyclin D1 expression level in HCC cells pretreated with SB203580 (for 6 h) or U0126 (for 24 h). Data are represented as mean±S.E.M. from three independent experiments. *P<0.05, **P<0.01, ***P<0.001

MAPK signaling pathway is known as one of the most common cellular signal transduction pathways involved in both physiologic processes and carcinogenesis. It has been reported that MAPK signaling activation can alter transcription of genes important in cell cycle, among which is cyclin D1.^26^ Therefore, to further investigate the function of MAPK signaling in MCM7–cyclin D1 pathway, we computed the correlation between *MCM7*, *CCND1* and *MAPK*-encoding genes using public microarray data (GSE21955). First, these data confirmed a significant positive correlation between the expression level of *MCM7* and *CCND1* in HCC cell lines (*r*^2^=0.479, *P*=0.018, Figure 4a). Second, the *MCM7* expression was shown to be correlated with almost all 11 MAPK-encoding genes (Figure 4b), suggesting a potential regulatory relationship between MCM7 and MAPK signaling pathway. To test our hypothesis, we screened the changes in the expression profiling of MAPK-encoding genes in MCM7-knockdown HCC cells. Our data showed that the expression of *extracellular regulated kinase 2* (*ERK2*), *ERK3*, *ERK4*, *ERK7*, *c-Jun N-terminal kinase 1* (*JNK1*), *JNK3* and *p38gamma* were suppressed by MCM7 downregulation in both HepG2 and SMMC-7721 cells (Figure 4c). In addition, the result of western blot assay confirmed that MCM7 downregulation significantly suppressed the levels of phosphorylated-ERK (p-ERK), p-JNK and p-p38 (Figures 4d and e).

When computing the correlation between *CCND1* and MAPK-encoding genes, we found that the expression of CCND1 was mainly associated with genes encoding ERK and p38, but not JNK (Figure 4f). After being treated with specific inhibitors that target ERK (U0126) and p38 (SB203580), cyclin D1 expression exhibited an approximately twofold decrease either in quiescent state or in platelet-derived growth factor (PDGF)-induced MAPK activation state (Figures 4g and h). It should be noted that the peak inhibitory effect of p38 inhibitor (SB203580) on cyclin D1 expression was reached at 6 h, whereas ERK inhibitor (U0126) treatment resulted in a delayed inhibitory effect (at 24 h) on cyclin D1 expression in HCC cell lines (Figure 4g).

### Inhibitory effect of MCM7 knockdown on HCC tumorigenicity *in vivo*

**Figure 5:**
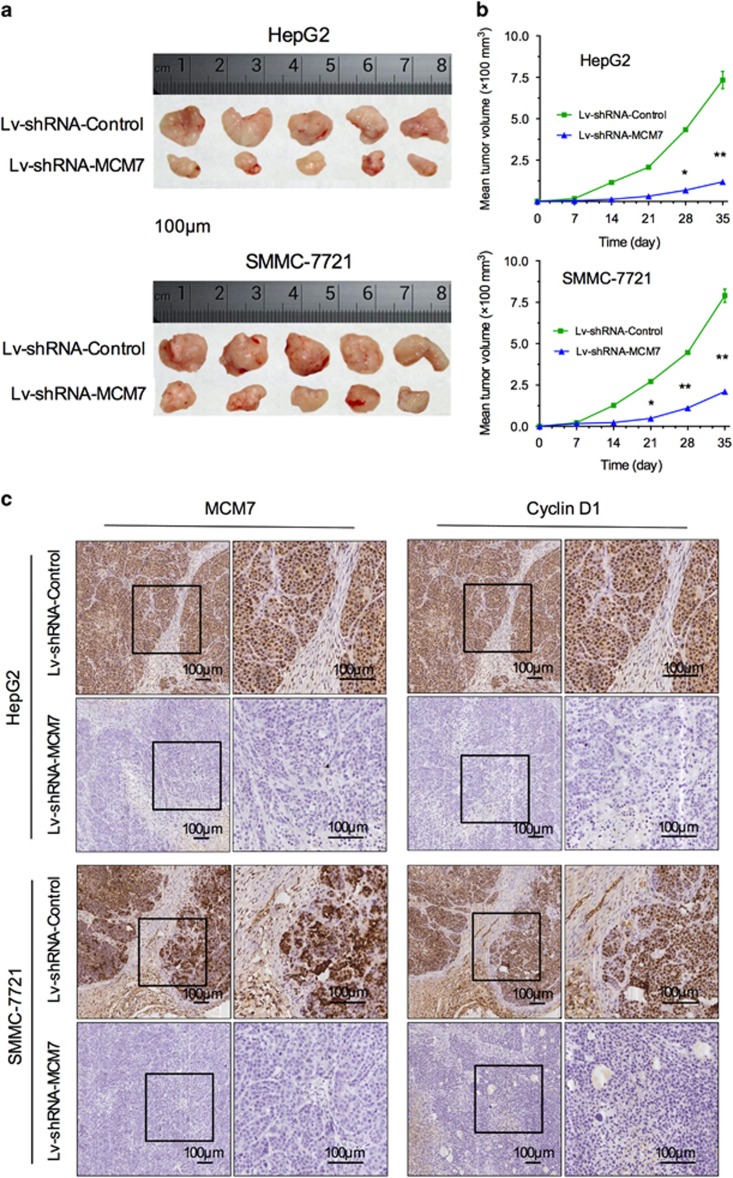
Knockdown of MCM7 inhibited tumor growth *in vivo*. (**a**) Tumor nodules after subcutaneously inoculation of HCC cells transfected with Lv-shRNA-MCM7 or Lv-shRNA-Control for 4 weeks (*n*=5 for each group). (**b**) Xenograft tumor volume of different groups bearing HCC cells transfected with Lv-shRNA-MCM7 or Lv-shRNA-Control. (**c**) Representative IHC staining of MCM7 and Cyclin D1 in xenograft tumors of different groups. Bar=100 *μ*m. *P<0.05, **P<0.01

MCM7 knockdown significantly inhibited cell proliferation via suppressing cyclin D1 expression in HCC cell lines, suggesting that MCM7 might be a potential therapeutic target for HCC. In this aspect, we further evaluated the anti-HCC effect of MCM7 knockdown in a xenograft mouse model. Nude mice were implanted subcutaneously with HCC cells that were transfected with either Lv-shRNA-MCM7 or Lv-shRNA-Control, and tumor size was measured once per week for 5 weeks. We found that the tumor size in Lv-shRNA-MCM7 group was much smaller than that of Lv-shRNA-Control group (Figure 5a). A significant tumor growth delay was observed in Lv-shRNA-MCM7 group as compared with controls (Figure 5b). To verify the inhibition of tumor growth *in vivo* was caused by MCM7 knockdown, we further detected the expression of MCM7 and cyclin D1 in those xenograft tumors. Our results showed that the expression levels of MCM7 and cyclin D1 was significantly reduced in Lv-shRNA-MCM7 group as compared with controls, indicating the involvement of functional MCM7–cyclin D1 pathway in HCC tumorigenesis (Figure 5c).

### Prognostic value of MCM7 and cyclin D1 in patients with HCC

**Figure 6:**
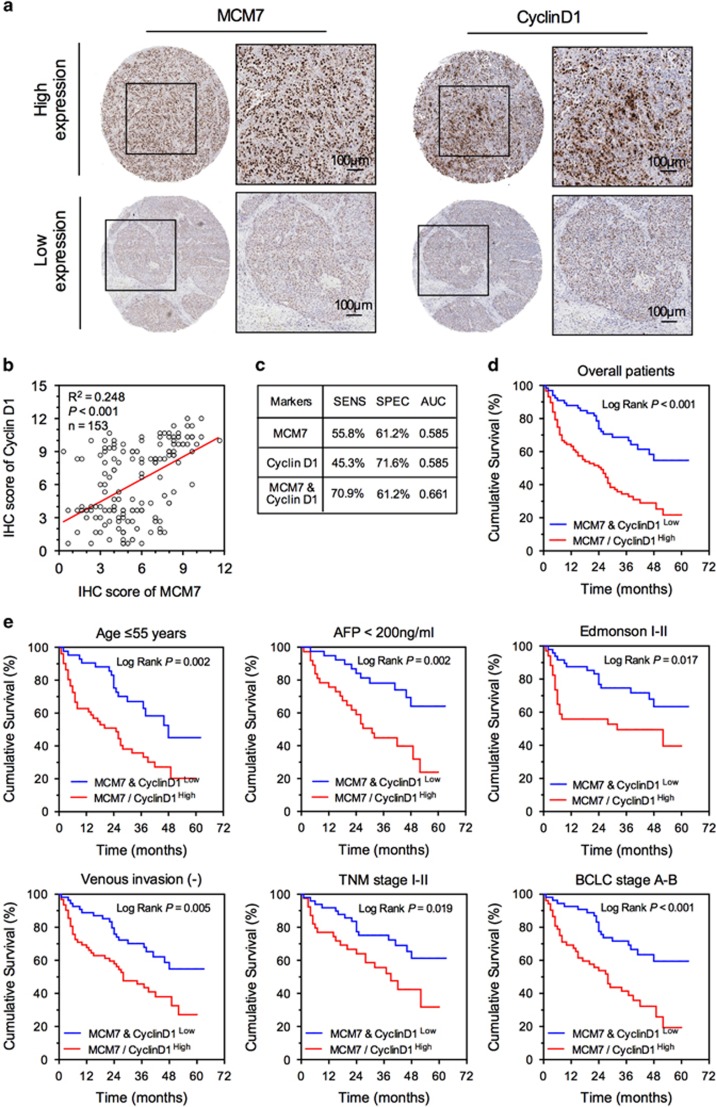
MCM7 and cyclin D1 served as potential biomarkers for prognosis of HCC. (**a**) Representative IHC staining of MCM7 and cyclin D1 in the same HCC tissue, bar=100 *μ*m. (**b**) Linear correlation analysis of MCM7 and cyclin D1 expression in HCC tumor tissues. (**c**) Diagnostic test evaluations of either MCM7 or cyclin D1 alone or in combination in predicting the risk for patients' death. Prognostic value of combination of MCM7 and cyclin D1 in overall HCC patients (**d**) and different subgroups (**e**)

The critical involvement of MCM7 and cyclin D1 in HCC pathology that was observed both *in vivo* and *in vitro* indicated that MCM7 and cyclin D1 might be potential prognostic biomarkers for HCC. To further evaluate their predictive values for patients' clinical outcome, the IHC assay on MCM7 and cyclin D1 was performed in 153 HCC patients. We found a significant positive correlation between MCM7 and cyclin D1 expression in HCC tumor tissues (Figures 6a and b) that confirmed the involvement of MCM7–cyclin D1 pathway in HCC. Compared with patients with both low MCM7 and low cyclin D1 expression, others were at a higher risk of death from HCC during the study (adjusted HR (95% CI): 2.57 (1.61–4.10), *P*<0.001) (Table 2 and Supplementary Table S2). Our data also demonstrated that the combination of MCM7 and cyclin D1 expression is a potential biomarker to predict mortality, having a relatively higher sensitivity (70.9%) than either MCM7 or cyclin D1 alone (Figure 6c). Based on the Kaplan–Meier survival curves, patients with MCM7^low^ and cyclin D1^low^ expression had a longer median OS time compared with those bearing either MCM7^high^ or cyclin D1^high^ expression (*P*<0.001, Figure 6d). In addition, combination of MCM7^low^ and cyclin D1^low^ expression was significantly associated with less aggressive characteristics, such as age ⩽55 years, AFP <200 ng/ml, Edmonson I–II, TNM stage I–II, BCLC stage A–B and negative venous invasion, suggesting that MCM7^low^ and cyclin D1^low^ expression can serve as novel biomarkers for HCC in relatively early-stage disease (Figure 6e).

## Discussion

In the present study, we found that high expression of MCM7 was associated with poor prognosis of the HCC patients. The upregulation of MCM7 enhanced the proliferation of HCC cells *in vitro* and tumorigenicity *in vivo*. Mechanistically, MCM7 upregulated cyclin D1 expression by modulating MAPK signaling pathway activity, thereby affecting cell cycle progression. Furthermore, a positive association between MCM7 and cyclin D1 expression was found in mouse model and human tumor tissues. Our data revealed that the combination of MCM7 and cyclin D1 had a highly prognostic value in HCC patients. Our studies presented robust evidence to support that MCM7 and its downstream target cyclin D1 are potential biomarkers for HCC prognosis.

Enabling replicative immortality is a hallmark of cancer.^7, 8^ DNA replication, the crucial central event during cell proliferation, is observed to be more pronounced in cancer cells than in normal cells. A number of proteins participating in DNA replication are abnormally expressed in cancer cells. For example, MCM7, a replication factor that binds to DNA double strand at replication origins in the late G1 phase and forms the pre-RC complex during cell cycle progression,^27^ is highly expressed in various cancers and can be used as a potential proliferation marker.^17, 21, 28, 29^ Zhou *et al.*^20^ reported that MCM7 was positive in 42 of 87 HCC tumors (48.2%) but negative in all noncancerous tissue. Consistently, we also observed that IHC index of MCM7 in HCC tumor was significantly higher than that in paired normal liver tissue. Although the positive immunostaining of MCM7 in cancer has been frequently observed, the oncogenic role of MCM7 in cancer development remains unclear. The first study on the molecular function of MCM7 was focused on genome analysis of prostate cancer.^17^ Researchers found that DNA copy number of MCM7 was increased several fold, and associated with prostate cancer relapse and metastasis. Subsequent studies have suggested that increases in MCM7 copy number and protein level were the driving force in skin and esophageal cancer models.^12, 30^ Liu *et al.*^31^ recently reported that knocking down of MCM7 inhibited proliferation and invasion of MHCC-97H cells (a HCC cell line with highly invasive potential). In this study, we further confirmed this result in both MCM7-knockdown HCC cells (HepG2 and SMMC-7721) and murine xenograft models. The mechanistic investigations revealed that knockdown of MCM7 resulted in the cell cycle arrest of HCC cells with the subsequent inhibition of tumor growth, suggesting that MCM7 might be a potential target for cancer therapy.

We identified cyclin D1 as a downstream target of MCM7 involved in cell cycle regulation. It has been widely accepted that cyclin D1 is a mitogenic sensor for the cell cycle machine and it also acts as an oncogene during cancer development.^32^ Overexpression of cyclin D1 and its accumulation in cancer cell nuclei can result from DNA damage and chromosomal abnormalities. Given the critical role of MCM7 in DNA replication licensing and causing chromosome instability,^33^ and the strong association between MCM7 and cyclin D1 expression in both human HCC tissue and murine xenograft tumor, it is reasonable to hypothesize that MCM7 acts as a regulator of cyclin D1. In fact, we previously reported that MCM7 depletion could prevent cyclin D1 expression in untransformed human dermal fibroblasts.^34^ In this study, as expected, we also found that knockdown of MCM7 significantly inhibited cyclin D1 expression in HCC cell lines, indicating an essential role of MCM7 in regulation of cell cycle.

Great efforts have been made over the past decade to explore the signaling pathways involved in the regulation of cyclin D1. The MAPK signaling pathway can induce multiple biologic events, such as cell proliferation, differentiation and survival, as well as stimulate cyclin D1 transcription in human cell lines.^35^ There are seven classes of MAPK signaling cascades, and three of them are essential for liver carcinogenesis, including ERK, JNK and p38 signaling cascades.^36^ Although Li *et al.*^37^ have observed simultaneous expression of MCM7, cyclin D1 and MAPKs in breast cancer cells, it was unclear whether MCM7 induced cyclin D1 expression through a MAPK-dependent mechanism in HCC. In the present study, inhibition of MCM7 expression suppressed the activities of all three MAPK signaling cascades. However, only two of them, ERK and p38, but not JNK signaling cascade, were found to be involved in the regulation of mitogen-induced cyclin D1 expression in HCC cells, consistent with previous studies.^35, 38^ A possible explanation for this observation is that JNK signaling cascade is generally responsible for the apoptotic response induced by DNA damage, but not the mitogen-induced cell proliferation.^39^ Taken together, the above evidence suggests that by activating MAPK pathways, especially the ERK and p38 signaling cascades, MCM7 may possess a significant role in the regulation of cyclin D1 and the subsequent cancer progression.

Accumulating evidence has supported MCM7 as a proliferation marker, comparable to the existing ones such as Ki-67 or proliferating cell nuclear antigen (PCNA) in multiple malignancies, including lymphoma,^14^ colorectal cancer,^15^ ovary cancer,^16^ prostate cancer^17^ and lung cancer.^18^ Here we confirmed that MCM7 expression could be an independent survival predictor for HCC. The higher MCM7 protein level was correlated with a worse prognosis. Interestingly, we found a statistically significant association between them only in patients with early stage or less aggressive cancer. Recently, Liu *et al.*^40^ evaluated the prognostic values of MCM7 in 494 patients with non-small-cell lung cancer (NSCLC). They found that patients with higher MCM7 expression level had a significant poorer OS compared with those with low MCM7 expression in early stages of NSCLC (*P*<0.00001). Similarly, Fujioka *et al.*^41^ also reported that MCM7 was related to poor prognosis in patients with stage I lung adenocarcinoma. A possible explanation for above findings is that MCM7, as a regulator controlling when DNA replication can begin, contributes to oncogene-driven tumorigenesis.^12^ Altered expression of MCM7 is to be considered as an early event during tumorigenesis. Higher MCM7 expression is always associated with high proliferative capacity in the early stage of cancer.^42^ In contrast, in late-stage cancer patients, many confounding factors, including tumor size, vascular or lymphocyte invasion, can affect the prognostic values of MCM7 that might produce a nonsignificant result.

Considering the regulatory relationship between MCM7 and cyclin D1, we further studied the prognostic value of the combination of MCM7 and cyclin D1 in HCC patients. As expected, the prognostic sensitivity of the combination was higher than either MCM7 or cyclin D1 alone. The moderate sample size in our study limited the validity of some stratified analyses. Although the diagnostic power for MCM7 and cyclin D1 in combination is rather modest, our present study offers evidence for the future investigation of MCM7 and cyclin D1 as prognostic markers in HCC management. Therefore, a large prospective validation study needs to be performed before their application in the clinic.

In summary, MCM7, a key factor involved in DNA replication initiation, was shown to be overexpressed in mostly HCC tumors and significantly associated with a poor prognosis of HCC patients. Mechanistically, MCM7 promotes HCC cell proliferation via upregulating MAPK–cyclin D1 pathway both *in vitro* and *in vivo*. In addition, our study indicates a joint effect of MCM7–cyclin D1 molecular markers in predicting the prognosis of HCC patients. Our data also shed light on the potential of MCM7 as a therapeutic target for HCC.

## Materials and Methods

### Patients and tissue samples

A total of 153 patients, including 101 males and 52 females, with an average age of 53.5 years (range: 24–80 years) were consecutively enrolled in this study. The clinicopathological characteristics of patients, including age, gender, hepatitis virus infection status, cirrhosis, Child–Pugh score, serum AFP level, tumor size, tumor number, tumor differentiation grade, TNM stage, capsule integrity and venous invasion are summarized in Supplementary Table S3. This research was approved by the institutional review board (IRB) of First Affiliated Hospital of Xi'an Jiaotong University.

### IHC assay

The expression levels of MCM7 or cyclin D1 in tissue samples were determined by using IHC assay. Briefly, tissue samples were fixed in 10% buffered formalin, embedded in paraffin and sectioned at 4 *μ*m thickness. Sections were stained with antibodies as described in other study.^43^ The detailed protocols and scoring system are shown in Supplementary Materials and Methods.

### Transduction of lentiviral vectors

Two HCC cell lines (HepG2 and SMMC-7721) were transfected using lentiviral vectors (Lv-shRNA-MCM7 and Lv-shRNA-Control) according to the manufacture's instruction (GenePharma Co., Shanghai, China). The detailed protocols are shown in Supplementary Materials and Methods.

### Western blotting and qRT-PCR assays

The intercellular expression levels of genes and proteins were determined by using western blotting and qRT-PCR methods, respectively. The detailed protocols are provided in Supplementary Materials and Methods. The primers used in qRT-PCR assay are listed in Supplementary Table S4. Antibodies used in this study were listed in Supplementary Table S5.

### MTT and colony formation assays

The *in vitro* cell proliferation of HCC cell lines was measured using the MTT method. Cell colony formation assay was used to assess the effect of MCM7 knockdown on the reproductive potential of HCC cells. The detailed protocols are provided in Supplementary Materials and Methods.

### Cell cycle and apoptosis analysis

Cell cycle progression was determined by flow cytometry of propidium iodide (PI)-stained cells on a flow cytometer (FACS Calibur, BD, Franklin Lakes, NJ, USA). Meanwhile, cell apoptosis was determined by flow cytometry of Annexin V-APC and PI (eBioscience, San Diego, CA, USA)-stained cells according to the manufacturer's instructions. The detailed protocols are provided in Supplementary Materials and Methods.

### Tumorigenicity assay *in vivo*

The protocol of tumorigenicity assay *in vivo* was approved by the institutional animal care and use committee (IACUC) at College of Medicine, Xi'an Jiao Tong University. The detailed protocols are provided in Supplementary Materials and Methods.

### Statistical analysis

Statistical analyses were performed using PASW Statistics 19 (SPSS Inc., Chicago, IL, USA). The detailed statistical methods used in this study are shown in Supplementary Materials and Methods. A *P-*value of ^<^0.05 was considered significant.

## Figures and Tables

**Table 1:** Stratified analysis on the association between MCM7 expression and overall survival of HCC patients

**Variable**	**Total number**	**MCM7**^**high**^		**MCM7**^**low**^		**HR (95% CI)**^a^	***P*****-value**^a^
		**Case number**	**MST (month)**	**Case number**	**MST (month)**		
Overall	153	74	26.0	79	46.0	1.64 (1.07–2.51)	**0.023**
*Age (years)*							
⩽55	93	41	26.0	52	38.0	1.44 (0.84–2.48)	0.182
>55	60	33	28.0	27	>63.0	2.00 (0.97–4.13)	0.062
							
*Gender*							
Male	101	51	22.0	50	>63.0	1.87 (1.07–3.29)	**0.029**
Female	52	23	29.0	29	38.0	1.47 (0.75–2.86)	0.264
							
*Hepatitis virus infection*							
Negative	43	21	34.0	22	>63.0	2.40 (0.90–6.42)	0.08
Positive	110	53	20.0	57	38.0	1.53 (0.94–2.46)	0.084
							
*Cirrhosis*							
Absent	55	25	49.0	30	>62.0	1.48 (0.64–3.43)	0.361
Present	98	49	15.0	49	37.0	1.74 (1.06–2.87)	**0.028**
							
*Child–Pugh score*							
A+B	142	69	28.0	73	48.0	1.62 (1.03–2.54)	**0.035**
C	11	5	17.0	6	29.0	2.93 (0.63–13.71)	0.173
							
*Serum AFP level (ng/ml)*							
<200	76	35	32.0	41	52.0	2.46 (1.23–4.92)	**0.011**
⩾200	77	39	15.0	38	25.0	1.22 (0.71–2.11)	0.468
							
*Tumor size (cm)*							
⩽5	99	45	29.0	54	>63.0	1.98 (1.11–3.54)	**0.021**
> 5	54	29	18.0	25	26.0	1.08 (0.57–2.04)	0.823
							
*Tumor number*							
Single	103	45	26.0	58	39.0	1.84 (1.08–3.13)	**0.024**
Multiple (⩾2)	50	29	31.0	21	41.0	1.25 (0.60–2.58)	0.550
							
*Capsule integrity*							
Yes	70	35	26.0	35	46.0	1.74 (0.94–3.24)	0.078
No	83	39	27.0	44	39.0	1.51 (0.84–2.73)	0.173
							
*Venous invasion*							
Negative	111	47	28.0	64	>63.0	1.92 (1.14–3.25)	**0.015**
Positive	42	27	10.0	15	18.0	0.44 (0.35–1.57)	0.440
							
*Differentiation*							
Edmonson I–II	82	31	52.0	51	>63.0	1.65 (0.83-3.29)	0.152
Edmonson III–IV	71	43	22.0	28	27.0	1.16 (0.66–2.02)	0.609
							
*TNM stage*							
I+II	112	48	28.0	64	>63.0	1.77 (1.04–3.00)	**0.036**
III	41	26	13.0	15	23.0	0.91 (0.43–1.92)	0.808
							
*BCLC stage*							
A–B	106	46	28.0	60	>63.0	2.03 (1.18–3.47)	**0.010**
C	47	28	18.0	19	16.0	0.84 (0.42–1.71)	0.632

Abbreviations: BCLC, Barcelona clinic liver cancer; CI, confidence interval; HR, hazard ratio; MST, median survival time

a HR and *P*-value were derived from univariate analysis. The significant *P-*values (<0.05) are in bold

**Table 2:** Stratified7+cyclin D1 expression and overall survival of HCC patients

**Variable**	**Total number**	**MCM7**^**high**^**or cyclin D1**^**high**^		**MCM7**^**low**^ **and cyclin D1**^**low**^		**HR (95% CI)**^a^	***P*-value**^a^
		**Case number**	**MST (month)**	**Case number**	**MST (month)**		
Overall	153	87	25.0	66	>63.0	2.57 (1.61–4.10)	**<0.001**
*Age*							
⩽55 years	93	51	25.0	42	48.0	2.43 (1.36–4.33)	**0.003**
> 55 years	60	36	24.0	24	>63.0	2.72 (1.22–6.06)	**0.015**
							
*Gender*							
Male	101	57	20.0	44	>63.0	2.70 (1.46–5.01)	**0.002**
Female	52	30	26.0	22	46.0	2.61 (1.26–5.39)	**0.01**
							
*Hepatitis virus infection*							
Negative	43	23	31.0	20	>63.0	3.73 (1.23–11.36)	**0.02**
Positive	110	64	17.0	46	46.0	2.37 (1.41–3.99)	**0.001**
							
*Cirrhosis*							
Absent	55	28	42.0	27	>62.0	2.45 (1.00–6.01)	0.051
Present	98	59	15.0	39	48.0	2.54 (1.46–4.40)	**0.001**
							
*Child–Pugh score*							
A+B	142	80	26.0	62	>63.0	2.53 (1.55–4.12)	**<0.001**
C	11	7	18.0	4	46.0	4.84 (0.57–40.86)	0.147
*Serum AFP level*							
<200 ng/ml	76	37	31.0	39	>63.0	3.02 (1.47–6.23)	**0.003**
⩾200 ng/ml	77	50	13.0	27	37.0	1.95 (1.05–3.60)	**0.034**
							
*Tumor size*							
⩽5 cm	99	50	28.0	49	>63.0	2.74 (1.49–5.05)	**0.001**
>5 cm	54	37	17.0	17	30.0	1.86 (0.88–3.94)	0.105
							
*Tumor number*							
Single	103	56	24.0	47	>63.0	3.21 (1.77–5.83)	**<0.001**
Multiple (⩾2)	50	31	28.0	19	46.0	1.58 (0.74–3.38)	0.235
							
*Capsule integrity*							
Yes	70	40	24.0	30	>63.0	2.60 (1.33–5.11)	**0.005**
No	83	47	25.0	36	48.0	2.47 (1.29–4.74)	**0.006**
							
*Venous invasion*							
Negative	111	53	28.0	58	>63.0	2.53 (1.47–4.36)	**0.001**
Positive	42	34	13.0	8	16.0	1.61 (0.56–4.62)	0.375
							
*Differentiation*							
Edmonson I–II	82	34	32.0	48	>63.0	2.25 (1.13–4.48)	**0.021**
Edmonson III–IV	71	53	22.0	18	37.0	1.82 (0.92–3.64)	0.088
							
*TNM stage*							
I+II	112	55	28.0	57	>63.0	2.53 (1.45–4.40)	**0.001**
III	41	32	13.0	9	26.0	1.60 (0.61–4.19)	0.337
							
*BCLC stage*							
A–B	106	52	28.0	54	>63.0	2.71 (1.55–4.75)	**0.001**
C	47	35	17.0	12	46.0	1.67 (0.69–4.06)	0.259

Abbreviations: CI, confidence interval; HR, hazard ratio; MST, median survival time

a HR and *P*-value were derived from univariate analysis. The significant *P-*values (<0.05) are in bold

## Abbreviations

MCM7: minichromosome maintenance complex component 7
HCC: hepatocellular carcinoma
OS: overall survival
HBV: hepatitis B virus
AFP: *α*-fetoprotein
IHC: immunohistochemistry
qRT-PCR: quantitative real-time reverse transcription PCR
CCND1: cyclin D1
RB: retinoblastoma protein
ERK: extracellular regulated kinase
JNK1: c-Jun N-terminal kinase 1
PDGF: platelet-derived growth factor
PCNA: proliferating cell nuclear antigen
NSCLC: non-small-cell lung cancer
PI: propidium iodide
IACUC: institutional animal care and use committee
IRB: institutional review board
