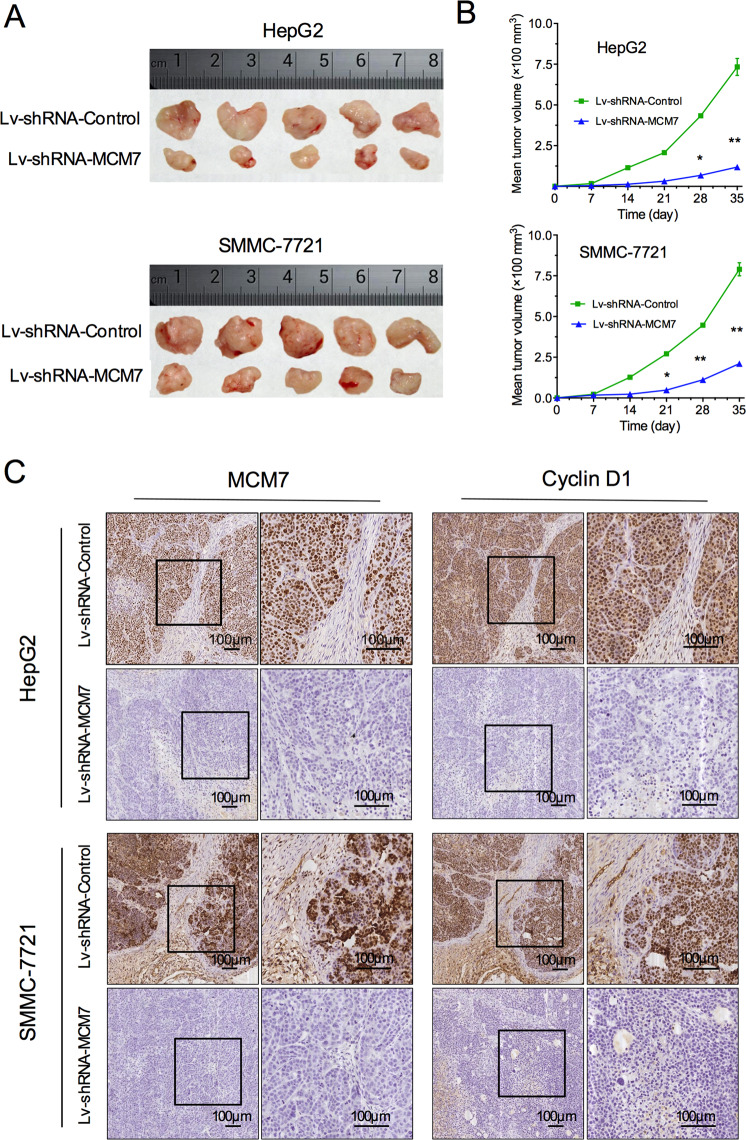# Correction to: MCM7 promotes cancer progression through cyclin D1-dependent signaling and serves as a prognostic marker for patients with hepatocellular carcinoma

**DOI:** 10.1038/s41419-022-05405-4

**Published:** 2022-11-10

**Authors:** Kai Qu, Zhixin Wang, Haining Fan, Juan Li, Jie Liu, Pingping Li, Zheyong Liang, Hongli An, Yina Jiang, Qiushi Lin, Xiaoqun Dong, Peijun Liu, Chang Liu

**Affiliations:** 1 Department of Hepatobiliary Surgery, The First Affiliated Hospital of Xi’an Jiaotong University, Xi’an, 710061 Shaanxi China; 2 Department of Hepatopancreatobiliary Surgery, Affiliated Hospital of Qinghai University, Xining, 810001 Qinghai China; 3 Center for Translation Medicine, The First Affiliated Hospital of Xi’an Jiaotong University, Xi’an, 710061 Shaanxi China; 4 Department of Pathology, The First Affiliated Hospital of Xi’an Jiaotong University, Xi’an, 710061 Shaanxi China; 5 Department of Internal Medicine, College of Medicine, The University of Oklahoma Health Sciences Center, Oklahoma City, OK 73104 USA

Correction to: *Cell Death and Disease* 10.1038/cddis.2016.352, published online 9 February 2017

The original version of this article contained an error in figure 5. The IHC staining of MCM7 was replaced by the figure of cyclin D1. The correct figure can be found below.