# Supplementary material for: MCM7 promotes cancer progression through cyclin D1-dependent signaling and serves as a prognostic marker for patients with hepatocellular carcinoma
Source: Cell Death Dis. 2017 Feb 9;8(2):e2603–. doi: 10.1038/cddis.2016.352 (PMC5386449; doi:10.1038/cddis.2016.352)
Supplement: Supplementary Figures [file cddis2016352x1.doc]

**Supplementary Figures**

**
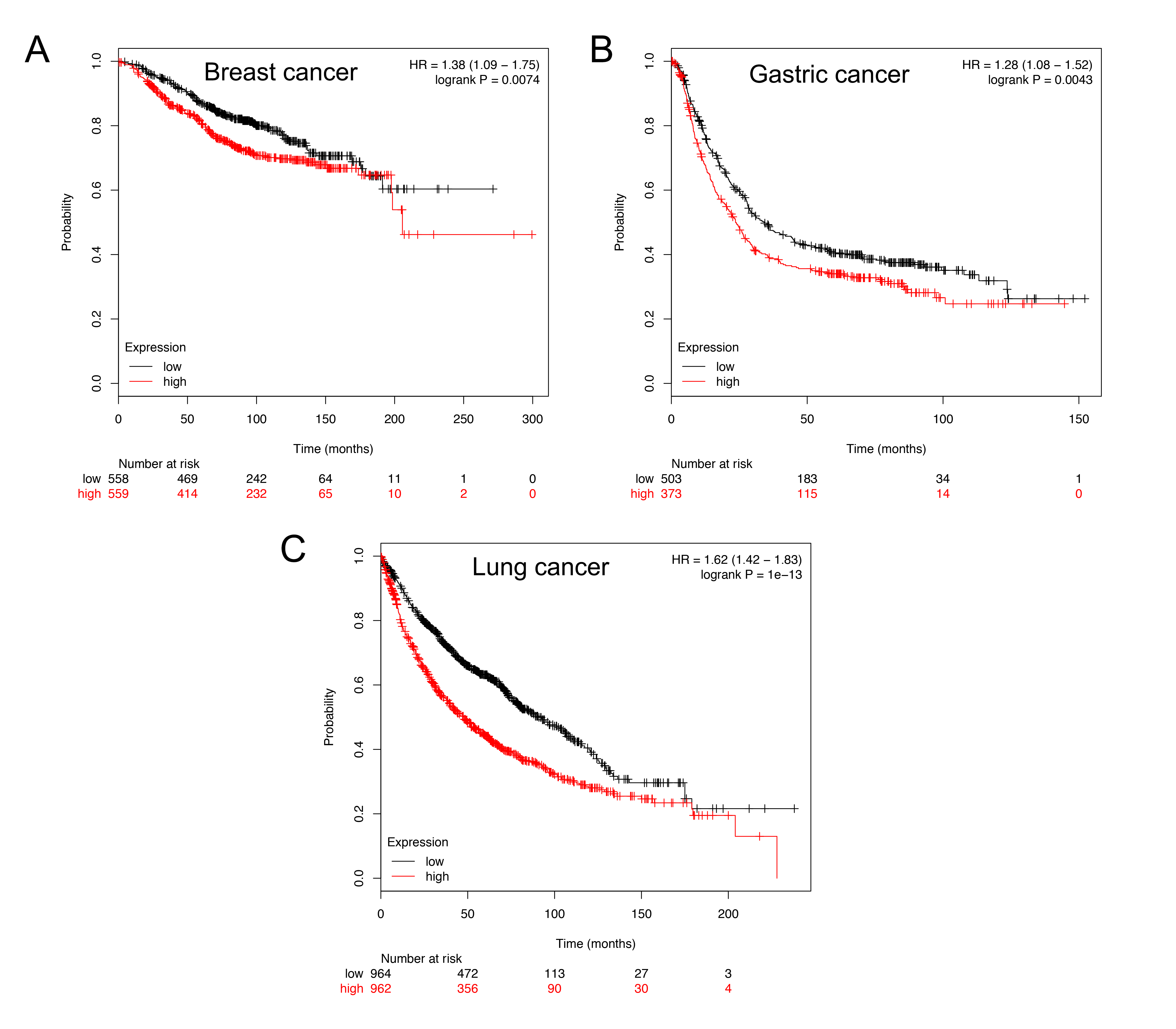
**

**Figure S1. High MCM7 expression was associated with worse overall survival in patients with breast cancer (A), gastric cancer (B) or lung cancer (C).** Data were obtained from http://kmplot.com/analysis/.
