# Supplementary material for: MCM7 promotes cancer progression through cyclin D1-dependent signaling and serves as a prognostic marker for patients with hepatocellular carcinoma
Source: Cell Death Dis. 2017 Feb 9;8(2):e2603–. doi: 10.1038/cddis.2016.352 (PMC5386449; doi:10.1038/cddis.2016.352)
Supplement: Supplementary Tables [file cddis2016352x2.doc]

**Supplementary Tables**

**Table S1. Univariate and multivariate analysis of factors associated with HCC overall survival**

| **Variable** | **Total number** | **Vital status, n (%)** | | **Crude HR**  **(95% CI)** | ***P*^a^** | **Adjusted HR**  **(95% CI)** | ***P*^b^** |
| --- | --- | --- | --- | --- | --- | --- | --- |
|  |  | **Dead** | **Alive** |  |  |  |  |
| Overall | 153 | 86 (56) | 67 (44) | - |  | - |  |
| Age (years) |  |  |  |  |  |  |  |
| ≤ 55 | 93 | 53 (57) | 40 (43) | 1 (reference) |  | 1 (reference) |  |
| > 55 | 60 | 33 (55) | 27 (45) | 0.90 (0.58-1.40) | 0.460 | 0.91 (0.59-1.41) | 0.678 |
| Gender |  |  |  |  |  |  |  |
| Female | 52 | 35 (67) | 16 (33) | 1 (reference) |  | 1 (reference) |  |
| Male | 101 | 51 (50) | 50 (50) | 1.27 (0.83-1.96) | 0.271 | 1.27 (0.83-1.96) | 0.279 |
| Hepatitis virus infection |  |  |  |  |  |  |  |
| Negative | 43 | 18 (42) | 25 (58) | 1 (reference) |  | 1 (reference) |  |
| Positive | 110 | 68 (62) | 42 (38) | **1.89 (1.12-3.19)** | **0.016** | **1.97 (1.14-3.43)** | **0.016** |
| Cirrhosis |  |  |  |  |  |  |  |
| Absent | 55 | 22 (40) | 33 (60) | 1 (reference) |  | 1 (reference) |  |
| Present | 98 | 64 (65) | 34 (35) | **2.14 (1.32-3.48)** | **0.002** | **2.33 (1.40-3.86)** | **0.001** |
| Child-Pugh score |  |  |  |  |  |  |  |
| A+B | 142 | 78 (55) | 64 (45) | 1 (reference) |  | 1 (reference) |  |
| C | 11 | 8 (73) | 3 (27) | 1.58 (0.76-3.29) | 0.530 | 1.55 (0.75-3.23) | 0.241 |
| Serum AFP level (ng/ml) |  |  |  |  |  |  |  |
| < 200 | 76 | 34 (45) | 42 (55) | 1 (reference) |  | 1 (reference) |  |
| ≥ 200 | 77 | 52 (68) | 25 (32) | **2.09 (1.35-3.23)** | **0.001** | **2.11 (1.36-3.28)** | **0.001** |
| Tumor size (cm) |  |  |  |  |  |  |  |
| ≤ 5 | 99 | 40 (40) | 59 (60) | 1 (reference) |  | 1 (reference) |  |
| > 5 | 54 | 46 (85) | 8 (15) | **2.01 (1.31-3.09)** | **0.001** | **1.99 (1.29-3.05)** | **0.002** |
| Tumor number |  |  |  |  |  |  |  |
| Single | 103 | 55 (53) | 48 (47) | 1 (reference) |  | 1 (reference) |  |
| Multiple (≥2) | 50 | 31 (62) | 19 (38) | 1.14 (0.73-1.78) | 0.556 | 1.13 (0.73-1.76) | 0.579 |
| Capsule integrity |  |  |  |  |  |  |  |
| Yes | 70 | 42 (60) | 28 (40) | 1 (reference) |  | 1 (reference) |  |
| No | 83 | 44 (53) | 39 (47) | 0.95 (0.62-1.46) | 0.829 | 0.95 (0.62-1.46) | 0.822 |
| Venous invasion |  |  |  |  |  |  |  |
| Negative | 111 | 56 (50) | 55 (50) | 1 (reference) |  | 1 (reference) |  |
| Positive | 42 | 30 (71) | 12 (29) | **2.07 (1.33-3.24)** | **0.001** | **2.03 (1.29-3.18)** | **0.002** |
| Differentiation |  |  |  |  |  |  |  |
| Edmonson I-II | 82 | 33 (40) | 62 (60) | 1 (reference) |  | 1 (reference) |  |
| Edmonson III-IV | 71 | 53 (75) | 5 (25) | **2.49 (1.60-3.88)** | **< 0.001** | **2.45 (1.57-3.83)** | **< 0.001** |
| TNM stage |  |  |  |  |  |  |  |
| I+II | 112 | 55 (49) | 57 (51) | 1 (reference) |  | 1 (reference) |  |
| III | 41 | 31 (76) | 10 (24) | **2.20 (1.41-3.42)** | **0.001** | **2.14 (1.37-3.36)** | **0.001** |
| BCLC stage |  |  |  |  |  |  |  |
| A-B | 106 | 54 (51) | 52 (49) | 1 (reference) |  | 1 (reference) |  |
| C | 47 | 32 (68) | 15 (32) | **1.88 (1.21-2.92)** | **0.005** | **1.84 (1.18-2.88)** | **0.007** |
| MCM7 expression |  |  |  |  |  |  |  |
| Low | 79 | 38 (48) | 41 (52) | 1 (reference) |  | 1 (reference) |  |
| High | 74 | 48 (65) | 26 (35) | **1.64 (1.07-2.51)** | **0.023** | **1.67 (1.09-2.57)** | **0.019** |

HR: hazard ratio; CI: confidence interval; AFP: alpha-fetoprotein; BLBC, Barcelona clinic liver cancer; MCM7: mini-chromosome maintenance protein 7.

^a^ *P*-value was derived from univariate analysis; ^b^ *P*-value was derived from multivariate analysis using Cox proportional hazards regression model. Adjusted hazard ratio was adjusted by age and gender. The HRs with significant *P* values (<0.05) were in bold.

**Table S2**. Stratified analysis on the association between (MCM7 + Cyclin D1) expression and overall survival of HCC patients

| **Variable** | **Total**  **number** | **MCM7^low^ and CyclinD1^low^** | | | **MCM7^low^ and CyclinD1^High^** | | | **MCM7^High^ and CyclinD1^low^** | | | **MCM7^High^ and CyclinD1^High^** | | |
| --- | --- | --- | --- | --- | --- | --- | --- | --- | --- | --- | --- | --- | --- |
|  |  | **Number** | **MST (m)** | **95%CI** | **Number** | **MST (m)** | **95%CI** | **Number** | **MST (m)** | **95%CI** | **Number** | **MST (m)** | **95%CI** |
| Overall | 153 | 66 | >63.0 | NA | 13 | 8.0 | 3.3-12.7 | 29 | 18.0 | 3.9-32.1 | 45 | 28.0 | 9.8-46.2 |
| Age |  |  |  |  |  |  |  |  |  |  |  |  |  |
| ≤ 55 years | 93 | 42 | 48.0 | NA | 10 | 6.0 | 1.4-10.6 | 17 | 14.0 | 4.6-23.4 | 24 | 28.0 | 13.6-42.4 |
| > 55 years | 60 | 24 | >63.0 | NA | 3 | 10.0 | 6.8-13.2 | 12 | 24.0 | 12.1-35.9 | 21 | 28.0 | 8.7-47.2 |
| Gender |  |  |  |  |  |  |  |  |  |  |  |  |  |
| Male | 101 | 44 | >63.0 | NA | 6 | 8.0 | 1.6-14.4 | 17 | 18.0 | 5.9-30.1 | 34 | 27.0 | 11.3-42.7 |
| Female | 52 | 22 | 46.0 | 34.0-58.0 | 7 | 6.0 | 3.4-8.6 | 12 | 14.0 | 0-47.9 | 11 | 28.0 | 6.6-49.4 |
| Hepatitis virus infection |  |  |  |  |  |  |  |  |  |  |  |  |  |
| Negative | 43 | 20 | >63.0 | NA | 2 | 10.0 | NA | 9 | 22.0 | 0-45.4 | 12 | >63.0 | NA |
| Positive | 110 | 46 | 46.0 | 33.6-58.4 | 11 | 8.0 | 4.8-11.1 | 20 | 15.0 | 4.0-26.0 | 33 | 26.0 | 14.0-38.0 |
| Cirrhosis |  |  |  |  |  |  |  |  |  |  |  |  |  |
| Absent | 55 | 27 | >63.0 | NA | 3 | 2.0 | NA | 10 | >63.0 | NA | 15 | 49.0 | 10.0-88.0 |
| Present | 98 | 39 | 48.0 | NA | 10 | 8.0 | 3.9-12.1 | 19 | 13.0 | 7.4-18.6 | 30 | 26.0 | 9.9-42.1 |
| Child-Pugh score |  |  |  |  |  |  |  |  |  |  |  |  |  |
| A+B | 142 | 62 | >63.0 | NA | 11 | 8.0 | 4.8-11.1 | 27 | 15.0 | 0-30.3 | 42 | 28.0 | 8.6-47.4 |
| C | 11 | 4 | 46.0 | NA | 2 | 25.0 | NA | 2 | 18.0 | NA | 3 | 5.0 | 0-11.4 |
| Serum AFP level |  |  |  |  |  |  |  |  |  |  |  |  |  |
| < 200 ng/ml | 76 | 39 | >63.0 | NA | 2 | 4.0 | NA | 12 | 24.0 | 8.7-39.3 | 23 | 49.0 | 2.3-95.7 |
| ≥ 200 ng/ml | 77 | 27 | 37.0 | 15.2-58.8 | 11 | 8.0 | 3.7-12.3 | 17 | 13.0 | 6.3-19.7 | 22 | 27.0 | 8.9-45.1 |
| Tumor size |  |  |  |  |  |  |  |  |  |  |  |  |  |
| ≤ 5 cm | 99 | 49 | >63.0 | NA | 5 | 8.0 | 1.6-14.4 | 17 | 22.0 | 8.6-35.4 | 28 | 52.0 | 10.5-93.5 |
| > 5 cm | 54 | 17 | 30.0 | 16.0-43.9 | 8 | 8.0 | 2.5-13.5 | 12 | 13.0 | 6.2-19.8 | 17 | 26.0 | 12.3-39.7 |
| Tumor number |  |  |  |  |  |  |  |  |  |  |  |  |  |
| Single | 103 | 47 | >63.0 | NA | 11 | 10.0 | 0-30.5 | 19 | 18.0 | 2.4-33.6 | 26 | 26.0 | 15.6-36.4 |
| Multiple (≥2) | 50 | 19 | 46.0 | 37.2-54.8 | 2 | 4.0 | NA | 10 | 15.0 | 1.1-28.9 | 19 | 49.0 | 11.5-86.5 |
| Capsule integrity |  |  |  |  |  |  |  |  |  |  |  |  |  |
| Yes | 70 | 30 | >63.0 | NA | 5 | 8.0 | 4.5-11.5 | 14 | 15.0 | 0-35.2 | 21 | 28.0 | 19.6-36.4 |
| No | 83 | 36 | 48.0 | NA | 8 | 10.0 | 0-36.3 | 15 | 18.0 | 1.6-34.4 | 24 | 28.0 | NA |
| Venous invasion |  |  |  |  |  |  |  |  |  |  |  |  |  |
| Negative | 111 | 58 | >63.0 | NA | 6 | 10.0 | 0-35.2 | 16 | 15.0 | 0-30.7 | 31 | 49.0 | 24.6-73.4 |
| Positive | 42 | 8 | 16.0 | NA | 7 | 6.0 | 0.9-11.1 | 13 | 18.0 | 0-38.0 | 14 | 16.0 | 0-37.9 |
| Differentiation |  |  |  |  |  |  |  |  |  |  |  |  |  |
| Edmonson I-II | 82 | 48 | >63.0 | NA | 3 | 5.0 | 0.2-9.8 | 7 | 32.0 | 0-101.3 | 24 | 52.0 | 10.2-93.8 |
| Edmonson III-IV | 71 | 18 | 37.0 | 20.4-53.6 | 10 | 10.0 | 0-36.3 | 22 | 15.0 | 4.7-25.3 | 21 | 26.0 | 16.3-35.7 |
| TNM stage |  |  |  |  |  |  |  |  |  |  |  |  |  |
| I+II | 112 | 57 | >63.0 | NA | 7 | 10.0 | 4.9-15.1 | 17 | 24.0 | 5.2-42.8 | 31 | 52.0 | 24.7-79.3 |
| III | 41 | 9 | 26.0 | 5.6-46.4 | 6 | 6.0 | 1.2-10.8 | 12 | 13.0 | 1.3-14.7 | 14 | 17.0 | 0-37.9 |
| BCLC stage |  |  |  |  |  |  |  |  |  |  |  |  |  |
| 0-B | 106 | 54 | >63.0 | NA | 6 | 10.0 | 0-35.2 | 15 | 15.0 | 3.6-26.4 | 31 | 49.0 | 24.6-73.4 |
| C-D | 47 | 12 | 46.0 | 2.0-90.0 | 7 | 6.0 | 0.9-11.1 | 14 | 18.0 | 0-47.3 | 14 | 17.0 | 0-37.9 |

MST (m), Median survival time (months); 95%CI, 95% of confidence interval; NA, not available.

**Table S3.** Demographic and clinical characteristics of HCC patients

| Variable | Number | Percentage |
| --- | --- | --- |
| All | 153 | 100 |
| Age (years) |  |  |
| ≤ 55 | 93 | 60.8 |
| > 55 | 60 | 39.2 |
| Gender |  |  |
| Male | 101 | 66 |
| Female | 52 | 34 |
| Hepatitis virus infection | |  |
| Negative | 43 | 28.1 |
| Positive | 110 | 71.9 |
| Cirrhosis |  |  |
| Absent | 55 | 35.9 |
| Present | 98 | 64.1 |
| Child-Pugh score | |  |
| A+B | 142 | 92.8 |
| C | 11 | 7.2 |
| Serum AFP level (ng/ml) | |  |
| < 200 | 76 | 49.7 |
| ≥ 200 | 77 | 50.3 |
| Tumor size (cm) | |  |
| ≤ 5 | 99 | 64.7 |
| > 5 | 54 | 35.3 |
| Tumor number | |  |
| Single | 103 | 67.3 |
| Multiple (≥ 2) | 50 | 32.7 |
| Capsule integrity | |  |
| Yes | 70 | 45.8 |
| No | 83 | 54.2 |
| Venous invasion | |  |
| Negative | 111 | 72.5 |
| Positive | 42 | 27.5 |
| Differentiation | |  |
| Edmonson I-II | 82 | 53.6 |
| Edmonson III-IV | 71 | 46.4 |
| TNM stage | |  |
| I+II | 112 | 73.2 |
| III | 41 | 26.8 |
| BCLC stage | |  |
| A-B | 106 | 69.3 |
| C | 47 | 30.7 |

**Table S4**. Primer sequences for qRT-PCR

| **Gene** | **Forward primer (5’--3’)** | **Reverse primer (5’--3’)** |
| --- | --- | --- |
| *MCM7* | CCTACCAGCCGATCCAGTCT | CCTCCTGAGCGGTTGGTTT |
| *CCND1* | GCTGCGAAGTGGAAACCATC | CCTCCTTCTGCACACATTTGAA |
| G1/S-regulatory genes |  |  |
| *ABL1* | TGAAAAGCTCCGGGTCTTAGG | TTGACTGGCGTGATGTAGTTG |
| *ANAPC2* | TATGTTGCGCGGAGTCTTGTT | GAAGCACCCATACAGACGCTG |
| *CCNE1* | AAGGAGCGGGACACCATGA | ACGGTCACGTTTGCCTTCC |
| *CDC25A* | CTCCTCCGAGTCAACAGATTCA | CAACAGCTTCTGAGGTAGGGA |
| *CDC34* | CATCGACTACCCATACTCTCCA | GAGAATGGTCCTGACGTTCTG |
| *CDC6* | ACCTATGCAACACTCCCCATT | TGGCTAGTTCTCTTTTGCTAGGA |
| *CDK4* | ATGGCTACCTCTCGATATGAGC | CATTGGGGACTCTCACACTCT |
| *CDK6* | CCAGATGGCTCTAACCTCAGT | AACTTCCACGAAAAAGAGGCTT |
| *CDKN1B* | TAATTGGGGCTCCGGCTAACT | TGCAGGTCGCTTCCTTATTCC |
| *CDKN3* | TCCGGGGCAATACAGACCAT | GCAGCTAATTTGTCCCGAAACTC |
| *CUL1* | GATCTGGGACGACCTCAGAG | CCCCTTTTTCGACTTAGAAGGAG |
| *CUL2* | TATGTGTGGCCTATCCTGAACC | TGCAAATGCCGAACATGATTTTC |
| *E2F1* | ACGTGACGTGTCAGGACCT | GATCGGGCCTTGTTTGCTCTT |
| *WEE1* | AGGGAATTTGATGTGCGACAG | CTTCAAGCTCATAATCACTGGCT |
| *SKP2* | ATGCCCCAATCTTGTCCATCT | CACCGACTGAGTGATAGGTGT |
| MAPK pathway genes |  |  |
| *ERK1* | CTACACGCAGTTGCAGTACAT | CAGCAGGATCTGGATCTCCC |
| *ERK2* | TACACCAACCTCTCGTACATCG | CATGTCTGAAGCGCAGTAAGATT |
| *ERK3* | CGGTGTCAATGGTTTGGTGC | GACGATGTTGTCGTGGTCCA |
| *ERK4* | GGTGACTTTGGTATGGCTCGT | CCAGAGGTCAATAGCCTGTGTA |
| *ERK7* | GGGCCTATGGCATTGTGTG | TCTCTGGGCATCTGTCTTATCC |
| *JNK1* | TCTGGTATGATCCTTCTGAAGCA | TCCTCCAAGTCCATAACTTCCTT |
| *JNK2* | GAAACTAAGCCGTCCTTTTCAGA | TCCAGCTCCATGTGAATAACCT |
| *JNK3* | CAGATGGAATTAGACCATGAGCG | TCAATGTGCAATCAGACTTGACT |
| *p38alpha* | TCAGTCCATCATTCATGCGAAA | AACGTCCAACAGACCAATCAC |
| *p38beta* | CTGAACAACATCGTCAAGTGCC | CATAGCCGGTCATCTCCTCG |
| *p38gamma* | GGGCTGCTGGACGTATTCAC | TGCCCATGAACGGCATCAC |
| *p38delta* | TGAGCCGACCCTTTCAGTC | AGCCCAATGACGTTCTCATGC |
| *GAPDH* | GGGGCTCTCCAGAACATCATCC | ACGCCTGCTTCACCACCTCTT |

qRT-PCR, quantitative reverse transcription polymerase chain reaction.

**Table S5. Primary antibodies for WB and IHC**

| **Antibody** | **Concentration**  **for WB** | **Concentration**  **for IHC** | **Specificity** | **Company** |
| --- | --- | --- | --- | --- |
| MCM7 | 1:500 | 1:200 | Mouse | Santa Cruz |
| Cyclin D1 | 1:500 | 1:200 | Mouse | Santa Cruz |
| CDK4 | 1:1000 | / | Mouse | Santa Cruz |
| p27 | 1:500 | / | Rabbit | Cell signaling |
| p21 | 1:500 | / | Mouse | Santa Cruz |
| RB | 1:200 | / | Mouse | Santa Cruz |
| p-RB | 1:400 | / | Rabbit | Cell signaling |
| ERK | 1:500 | / | Rabbit | Cell signaling |
| p-ERK | 1:500 | / | Rabbit | Cell signaling |
| JNK | 1:1000 | / | Rabbit | Cell signaling |
| p-JNK | 1:1000 | / | Mouse | Cell signaling |
| p38 | 1:1000 | / | Rabbit | Cell signaling |
| p-p38 | 1:1000 | / | Rabbit | Cell signaling |
| β-actin | 1:2000 | / | Rabbit | Santa Cruz |

Abbreviations: WB, western blot; IHC, immunohistochemistry.
