# Supplementary material for: MCM7 promotes cancer progression through cyclin D1-dependent signaling and serves as a prognostic marker for patients with hepatocellular carcinoma
Source: Cell Death Dis. 2017 Feb 9;8(2):e2603–. doi: 10.1038/cddis.2016.352 (PMC5386449; doi:10.1038/cddis.2016.352)
Supplement: Supplementary Methods [file cddis2016352x3.doc]

**Supplementary Materials and Methods**

***Inclusion criteria and information collection***

Patients underwent tumor resection for HCC without receiving any other anti-cancer therapy at the First Affiliated Hospital of Xi'an Jiaotong University, Shaanxi, China, between January 2008 and June 2010, were screened. The cases were selected based on the following criteria: (1) histologically proven primary HCC with available biopsy specimens; (2) no previous malignant diseases or second primary tumors; (3) no previous anti-cancer treatment or severe complications; and (4) no distant metastases. Surgical resection was defined as removal of all recognizable tumor tissue with negative microscopic margins. Diagnosis of HCC was histologically confirmed after surgical resection. Histologic grading of HCC was based on the criteria proposed by Edmonson and Steiner. Well-differentiation was defined as the grade 1 or 2, whereas poor-differentiation as the grade 3 or 4. Tumor clinical stage was defined according to the tumor-node-metastasis (TNM) classification system. We defined stage I and II cancer as early-stage, whereas stage III and IV as late-stage. All patients were followed up by the researchers until their death or the date of the last contact after discharge. The survival data were obtained via a median follow-up of 840 days (range: 30-1890 days). Written informed consent was obtained on the enrollment from each patient for the research use of his/her specimens. All procedures were conducted according to the guidelines approved by the Ethics Committee at First Affiliated Hospital of Xi'an Jiaotong University.

***Scoring system for immunohistochemistry***

Each archival specimen was formalin-fixed paraffin-embedded (FFPE) and then incised into continuous sections (4 µm thick) and deparaffinized with xylene. Activity of endogenous peroxidase was blocked by immersing the tissue sections in absolute methanol containing 0.3% hydrogen peroxidase for 30 minutes. The sections were pretreated with citrate buffer (pH = 6.0) for 20 minutes at 100ºC in a pressure cooker, and then blocked by serum for 30 minutes. After that, the sections were incubated overnight at 4ºC with monoclonal antibody against MCM7 (1:300; sc-9966, Santa Cruz, USA) or monoclonal antibody against cyclin D1 (1:1000; ab16663, Abcam, USA). After incubation with avidin-biotin complex (SP-9000, ZSGB-BIO, CHINA) for 20 minutes, samples were developed with 3, 3’-diaminobenzidine tetrahydrochloride. Finally, samples were counterstained with haematoxylin and mounted on slides. IHC images were obtained using a section microscope scanner (Leica MP, SCN400, Germany) equipped with Leica DFC Cameras-Image Acquisition System (software V3.5.0, Switzerland). The expression levels of MCM7 or cyclin D1 were assessed by the percentage of positively stained cells and the IHC index (IHCI) determined on the basis of the staining intensity and the percentage of immuno-reactive cells. Staining intensity was rated as 0 (negative), 1 (weakly positive), 2 (moderately positive), or 3 (strongly positive) and the percentage of staining was rated as 0 (0%), 1 (1-25%), 2 (26-50%), 3 (51-75%), or 4 (76-100%). The IHCI for each sample was calculated as the mean value of the products of the intensity scores and the respective corresponding percentage scores from three fields in the image of the sample. The expression level was considered as “low” if the IHCI was within the range of 1 to 6, and as “high” if the IHCI was between 7 and 12.

***Cell culture***

Human HCC cell line HepG2 was purchased from American Type Culture Collection (Manassas, VA, USA). SMMC-7721 cell line was kindly provided by the Molecular Biology Center of the First Affiliated Hospital, Xi’an Jiaotong University. Cells were cultured in DMEM (Invitrogen) supplemented with heat-inactivated 10% fetal bovine serum (Invitrogen) and antibiotics (penicillin and streptomycin) at 37 °C in a humidified incubator containing 5% CO_2_.

***Western blotting***

Cells and human samples were lysed in ice-cold RIPA lysis buffer (1% NP-40, 0.1% SDS, 0.5% sodium deoxycholate, 150 mmol/L NaCl and 10 mmol/L Tris-HCl) containing a protease inhibitor cocktail. The total protein concentration was determined using a Bio-Rad protein assay reagent (Bio-Rad, Hercules, CA, USA). Equivalent amounts of proteins (30 μg) were then separated by 12% SDS-PAGE and transferred to nitrocellulose membranes (Bio-Rad, Hercules, CA, USA). After being blocked in Tris buffered saline (TBS) containing 5% non-fat milk, the membranes were incubated with primary antibodies at 4 ºC for 12 h and then with horseradish peroxidase (HRP) conjugated anti-goat or anti-rabbit antibody (Zhongshan, Beijing, China) at a dilution of 1:3000 at room temperature for 1 h. Signals were detected on X-ray film using the ECL detection system (Pierce, Rockford, IL, USA). Equal protein loading was assessed by the expression of β-actin.

***Quantitative real time PCR (qRT-PCR)***

Total RNA was isolated from cells using the RNAfast200 Kit (Fastagen Biotech, Shanghai, China). Reverse transcription was performed using the PrimeScript® RT reagent Kit (TaKaRa Biothechnology, Dalian, China). qRT-PCR reactions were performed using a Bio-Rad CFX96 system (Bio-Rad Labs,Hercules, CA, USA) with SYBR Green PCR Master Mix (TaKaRa Biotech, China). The cycling parameters were denaturation at 95ºC for 15 s, annealing at 55ºC for 15 s and collection fluorescence at 72ºC for 15s. A melting-curve analysis was then performed to check the specificity of PCR. The mRNA expression was assayed in triplicate and normalized to the *β-actin* mRNA level. The relative levels were calculated using the Comparative-Ct Method (ΔΔCt method).

***Transduction of lentiviral vectors***

HepG2 and SMMC-7721 cells (2×10^6^ cells/mL) were transfected with lentiviral vectors at an MOI of 10, respectively. An empty vector was used as control group. After transduction, cells were seeded in 10-cm plates and grew in medium supplemented with 1 mg/mL of G418 for 14 d. Followed by discarding the transduction reagent, cells were re-seeded in 96-well plates at a low density to generate single-cell-derived colonies. A single-cell clone was transferred into a 24-orifice plate and allowed to expand, and the resulting cells were screened by G418 (500 μg/mL) for one month.

***MTT assay***

Cells were seeded into 96-well plates and transfected. During the indicated time periods, 0.1 ml of spent medium was replaced with an equal volume of fresh medium containing MTT 0.5 mg/ml. Plates were incubated at 37 ºC for 4 h, and then the medium was replaced by 0.1 ml of DMSO (Sigma, St Louis, MO, USA) and plates were shaken at room temperature for 10 min. The absorbance was measured at 490 nm.

***Colony formation assay***

Cells were transfected by lentiviral vectors and collected by trypsinization. Cells were reseeded in 100 mm cell culture dishes with 200 cells per dish and cultured in soft agar for 10 days. Then, cells were fixed using 4% paraformaldehyde and stained with 0.5% crystal violet. The abilities to form colonies of cells were evaluated by the colony formation number.

***Cell cycle analysis***

Forty-eight hours after transduction, cells were trypsinized, collected and washed twice with PBS. Then the cells were fixed with cold 70% ethanol for at least 1 h at 4 ºC. Before flow cytometric analysis, the cells were incubated with RNase at 0.01 mg/mL and PI at 50 µg/mL in the dark at room temperature for 30 min. A total of 10^4^ cells were analyzed for each sample. Cell cycle analysis was performed by Cell Quest software (BD, Franklin Lakes, NJ, USA). Experiments were done in triplicate.

***Tumorigenicity assay in vivo***

Male athymic nude mice (5-6 week-old) were purchased from the Laboratory of Animal Breeding and Research Center, Xi’an, China. To evaluate the role of MCM7 in tumor formation in vivo, we propagated HepG2 and SMMC-7721 cell pools using Lv-shRNA-MCM7 and Lv-shRNA-Control, and inoculated 1×10^6^ cells subcutaneously into mice (n = 5 for each group). Tumorigenicity was evaluated at 5 weeks after transplantation. Tumor volume and body weight were recorded per-5 days. The tumor size was calculated using the following formula: volume (mm3) =W2×L×0.5. Where W and L referred to the smaller and the larger diameter at each measurement. Mice were sacrificed by euthanasia at the end of the 5-week study period.

***Statistical analysis***

Paired *t*-test was used to compare the differences between the level of MCM7 expression in cancer and corresponding precancerous tissues. Associations between protein expressions and clinicopathological variables were assessed by the Mann-Whitney test. The association between MCM7 and cyclin D1 expression was evaluated by Spearman's rank correlation and linear regression. Cumulative survival curves were obtained by the Kaplan-Meier method and analyzed by the log-rank test. The overall survival (OS) was calculated from the date of diagnosis to the date of death or last follow-up. The disease-free survival (DFS) was calculated from the date of surgery to the date of first recurrence or progression of cancer. (The data of the patients alive at the date of last contact were censored). Univariate and multivariate Cox regression analyses were performed to identify independent risk factors that had significant impacts on patients’ survival. The hazard ratios (HRs) and 95% confidence intervals (CIs) of the prognostic factors were calculated.
